# Supplementary material for: Biallelic VARS variants cause developmental encephalopathy with microcephaly that is recapitulated in vars knockout zebrafish
Source: Nat Commun. 2019 Feb 12;10:708. doi: 10.1038/s41467-018-07953-w (PMC6372652; doi:10.1038/s41467-018-07953-w)
Supplement: Supplementary file 3 — Description of Additional Supplementary Files [file 41467_2018_7953_MOESM3_ESM.pdf]

Description of Additional Supplementary Files

File Name: Supplementary Data 1
Description: Detailed clinical information on all patients with bi-allelic VARS variants.

File Name: Supplementary Data 2
Description: A series of coronary sections from paraffin-embedded brains of 1-5 dpf vars+/+, vars+/- and vars-/- larvae. For each group a representative larva was chosen.
