# Supplementary material for: Biallelic VARS variants cause developmental encephalopathy with microcephaly that is recapitulated in vars knockout zebrafish
Source: Nat Commun. 2019 Feb 12;10:708. doi: 10.1038/s41467-018-07953-w (PMC6372652; doi:10.1038/s41467-018-07953-w)

1 dpf *vars+/+*

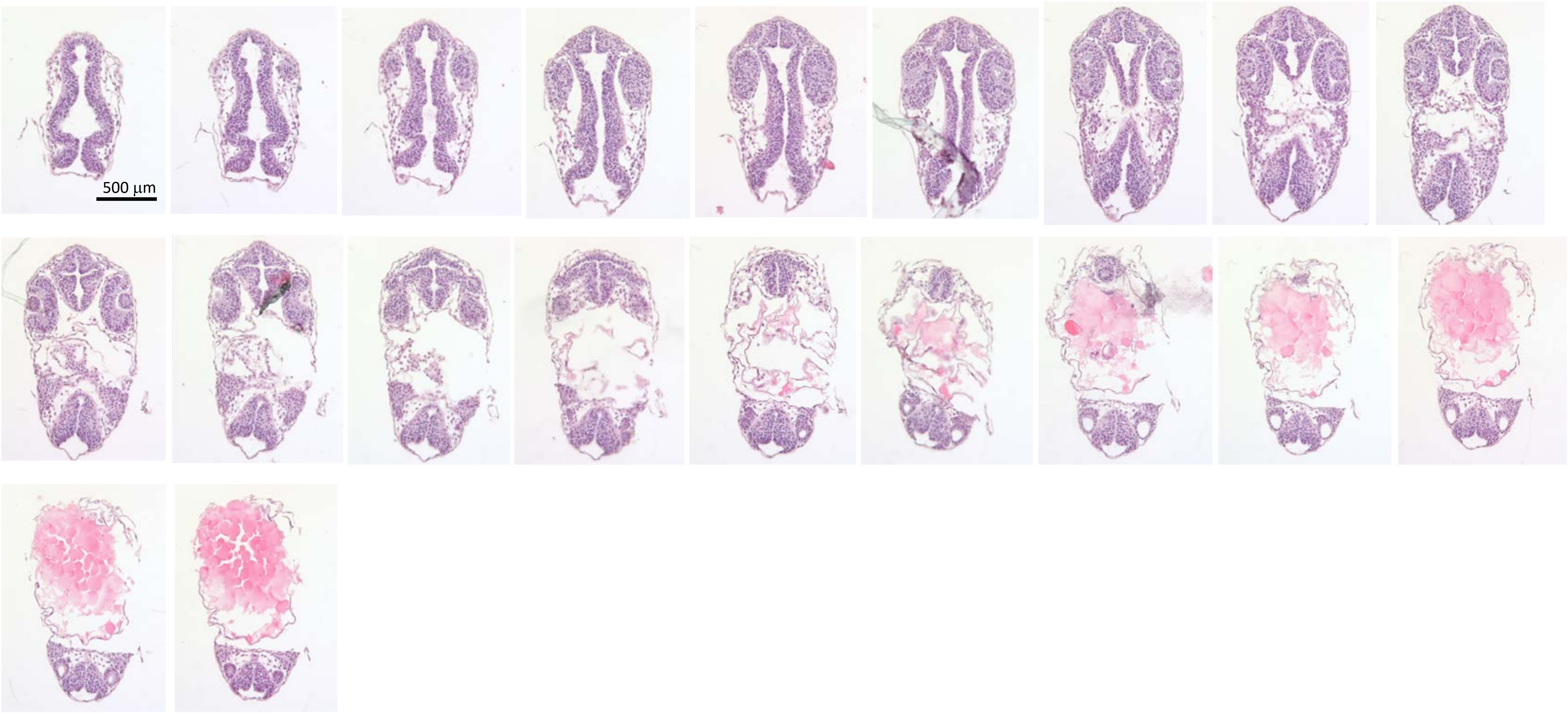

1 dpf *vars+/-*

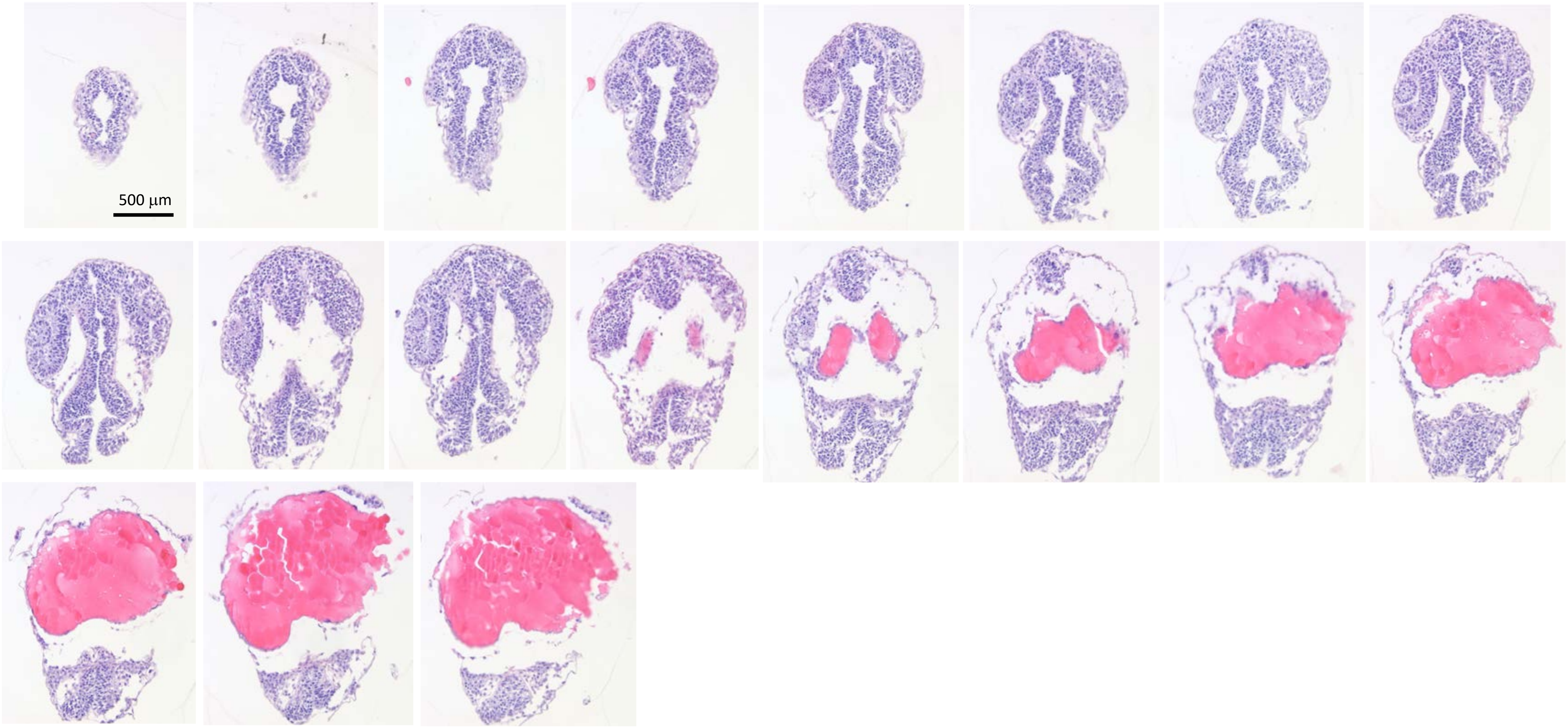

1 dpf *vars-/-*

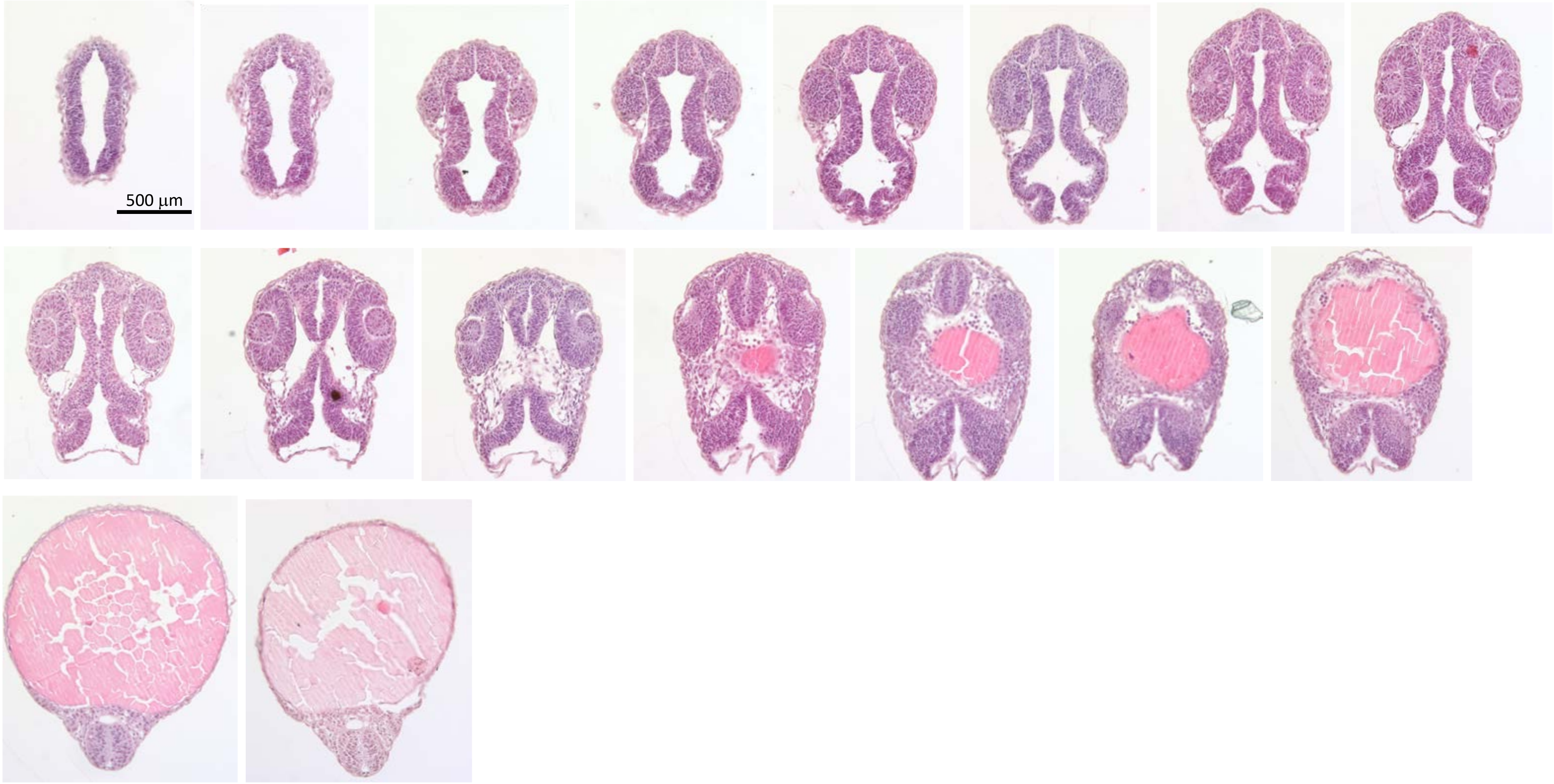

2 dpf *vars+/+*

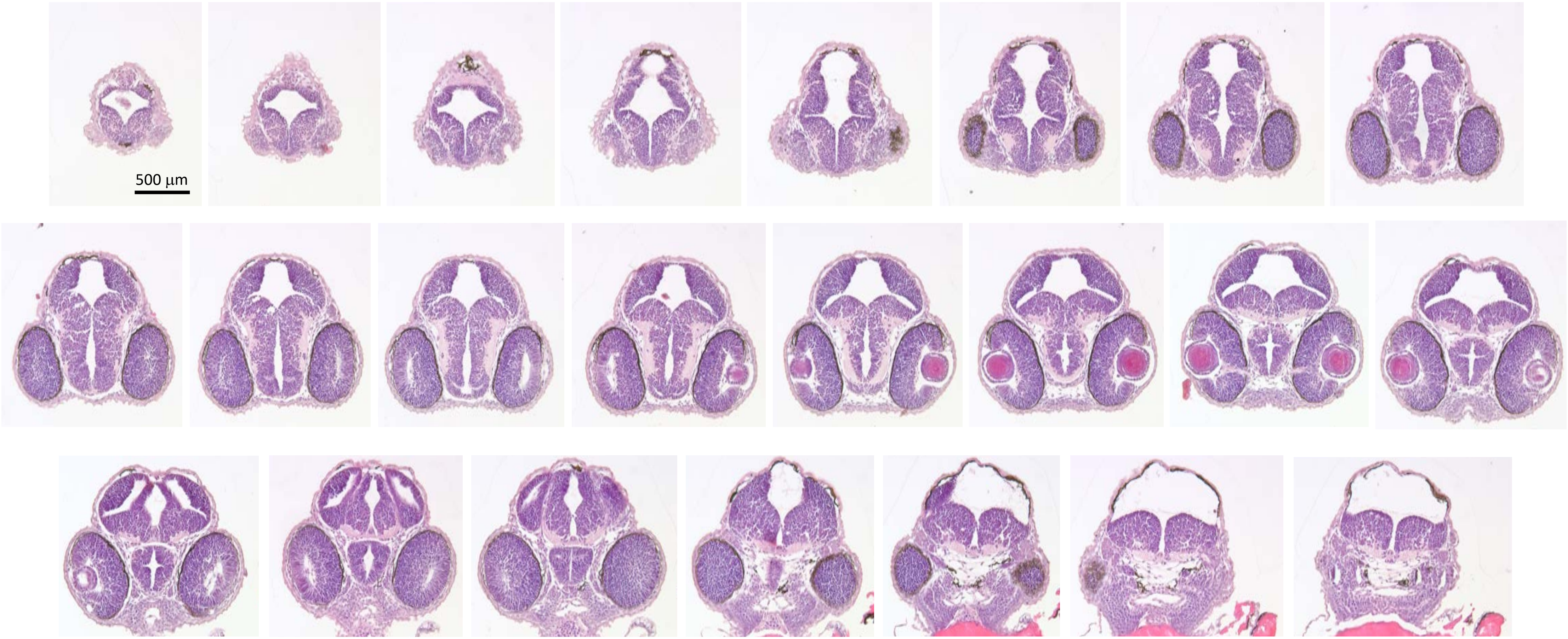

2 dpf *vars+/-*

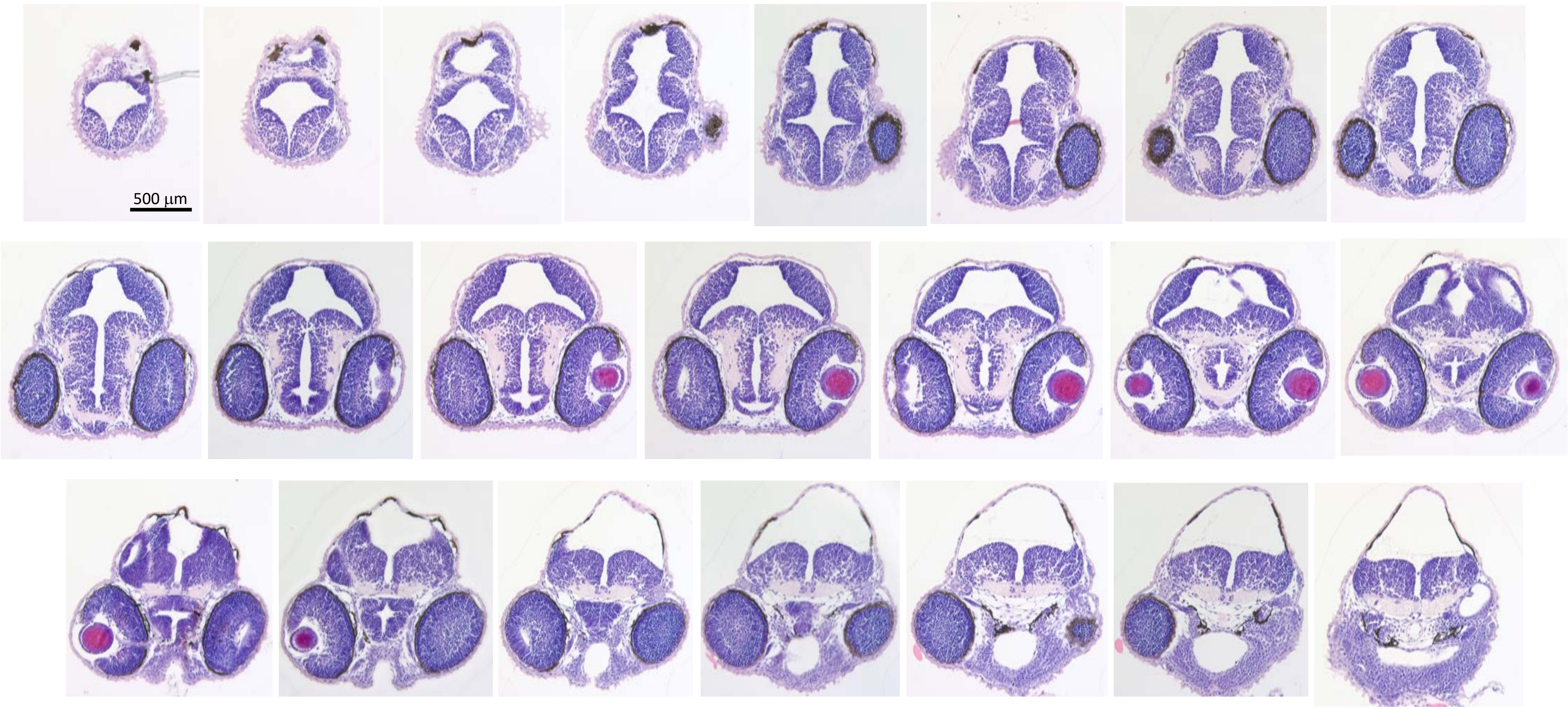

2 dpf *vars-/-*

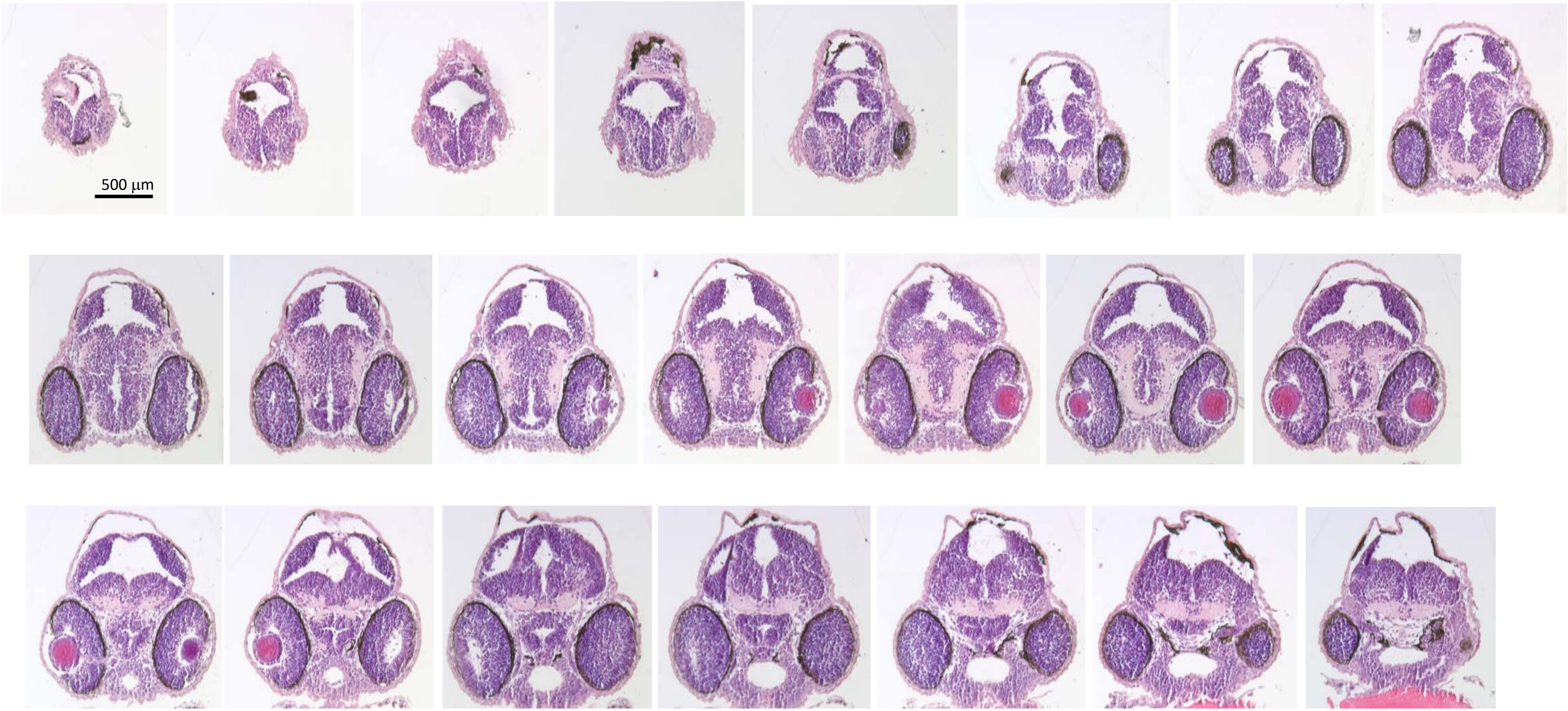

3 dpf *vars+/+*

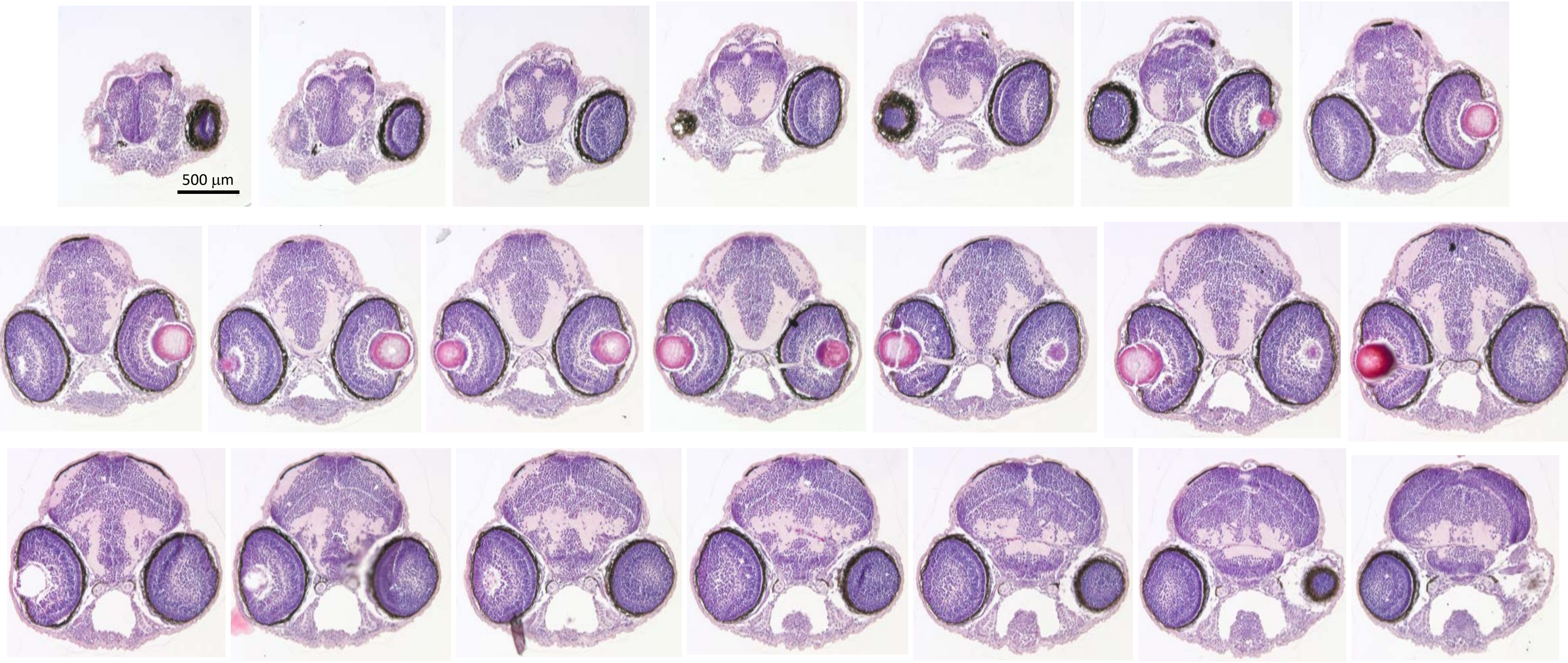

3 dpf *vars+/-*

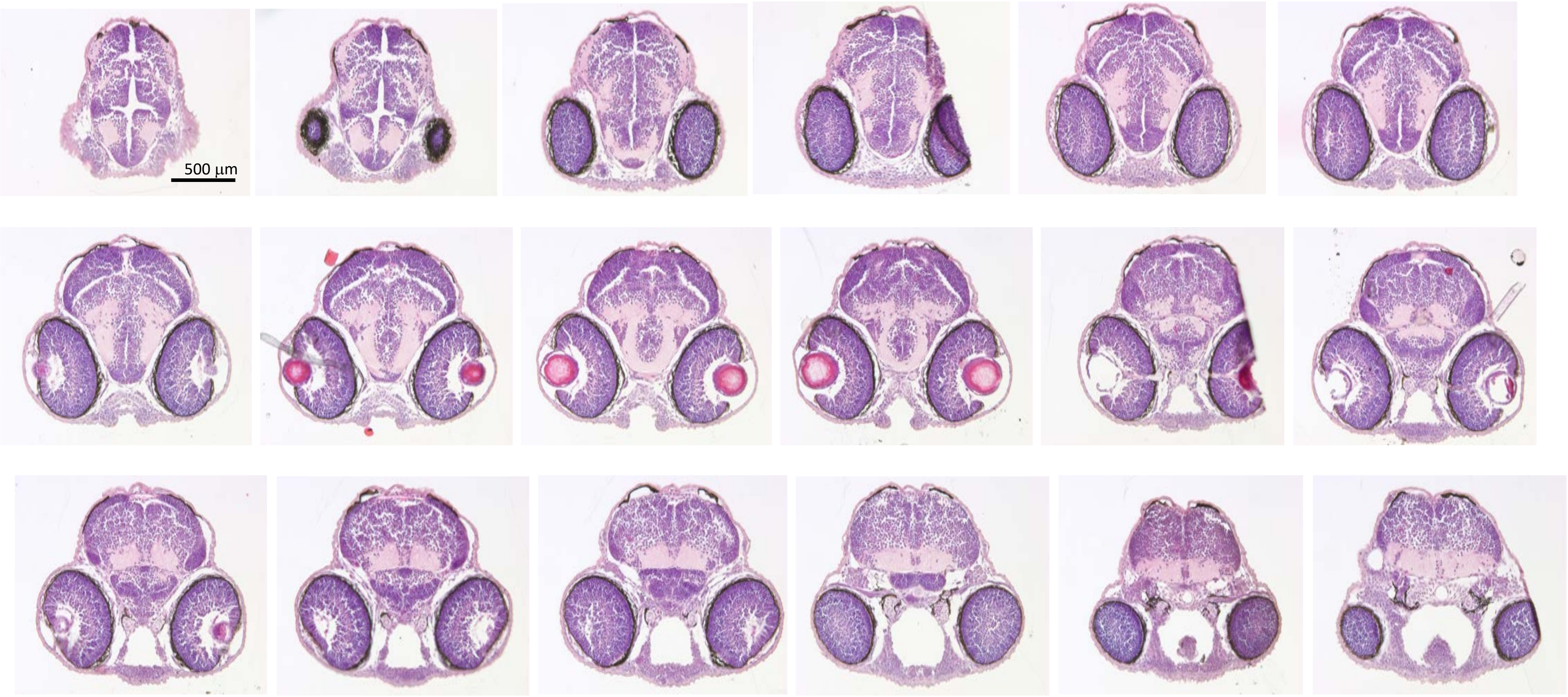

3 dpf *vars-/-*

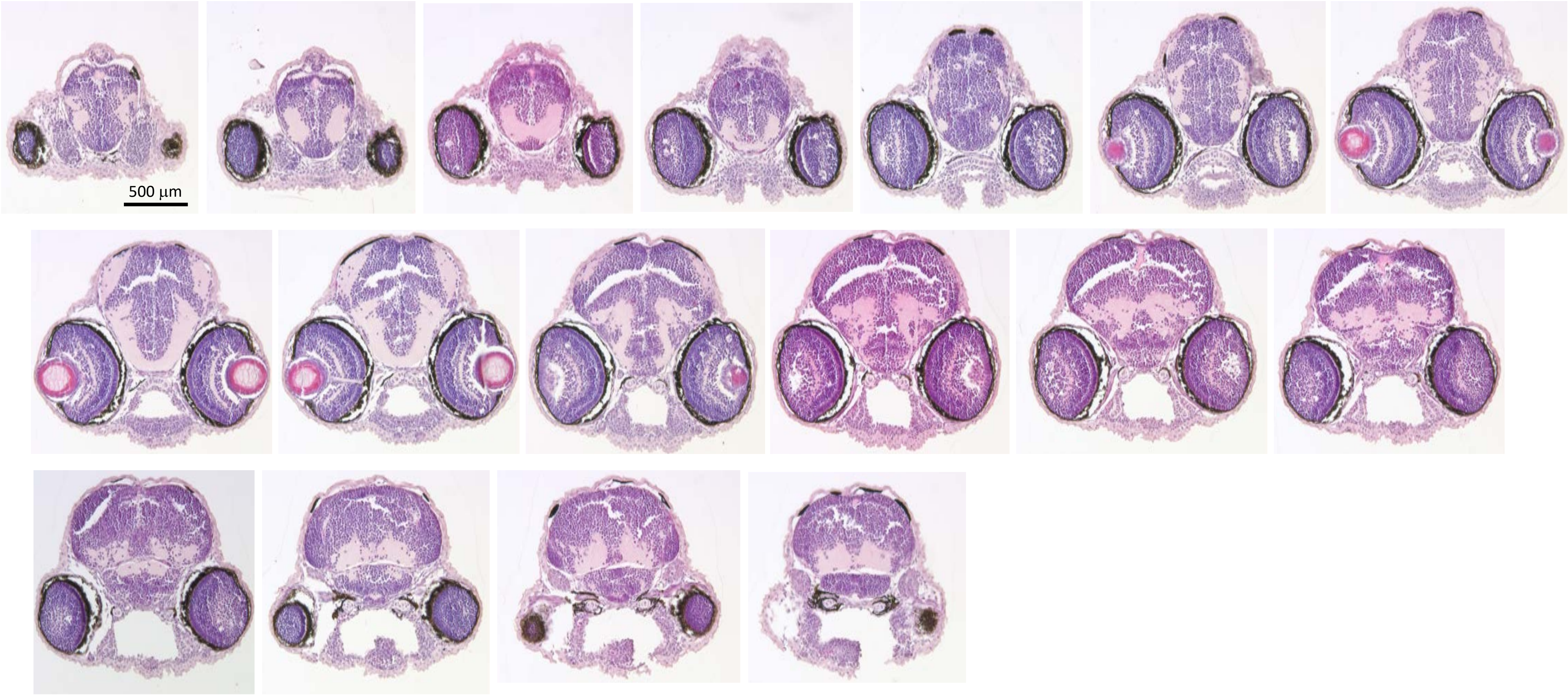

4 dpf *vars+/+*

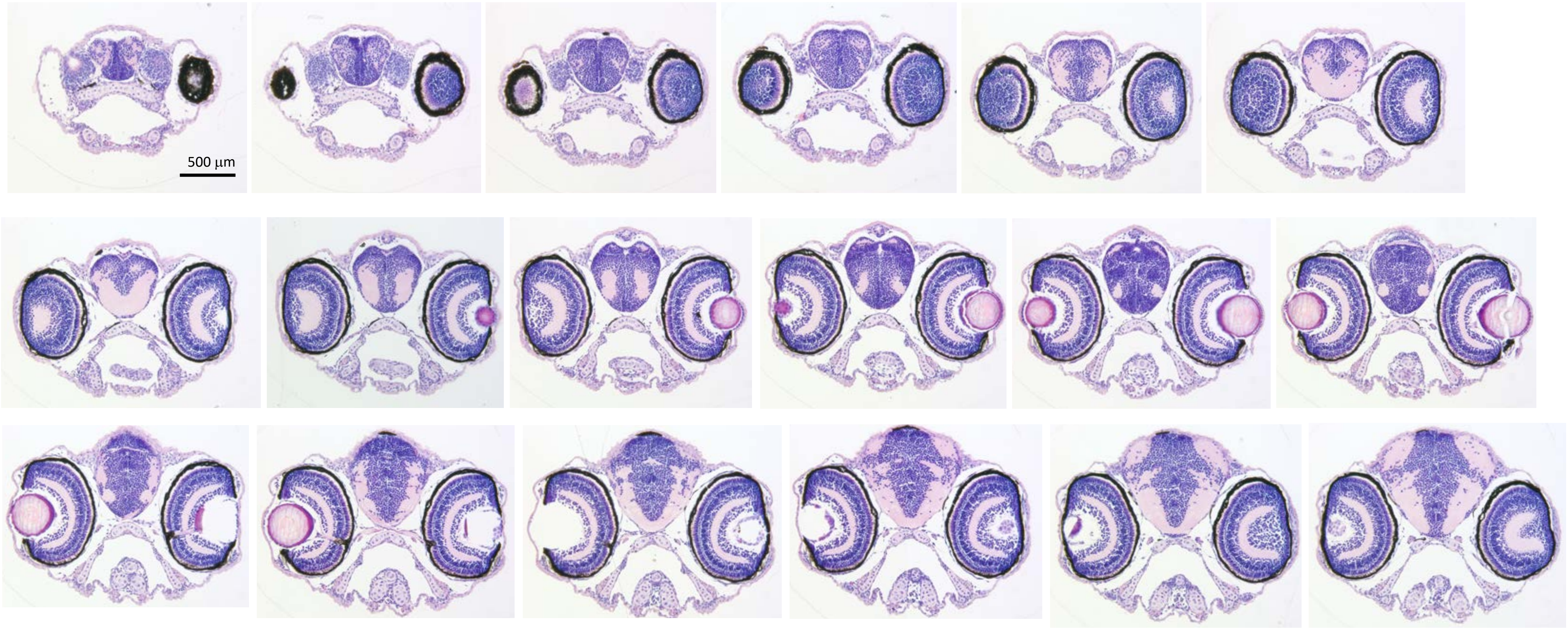

4 dpf *vars+/-*

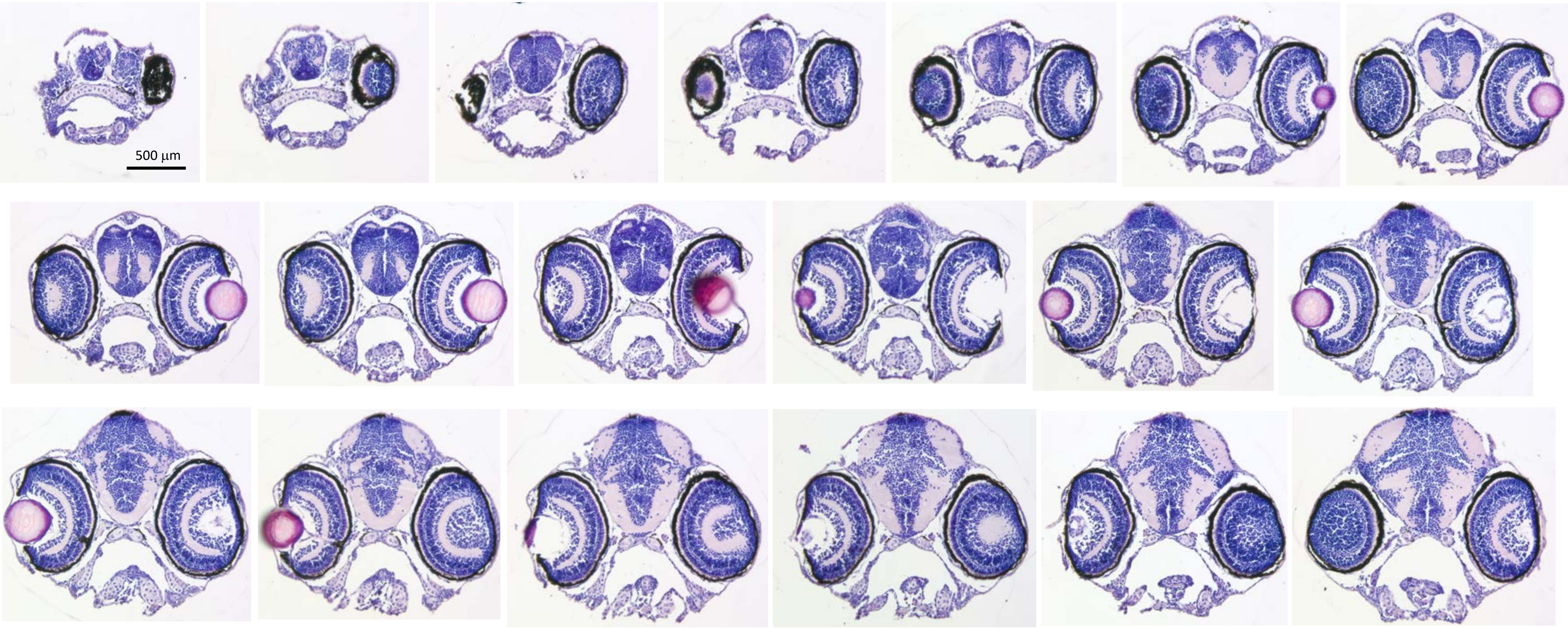

4 dpf *vars-/-*

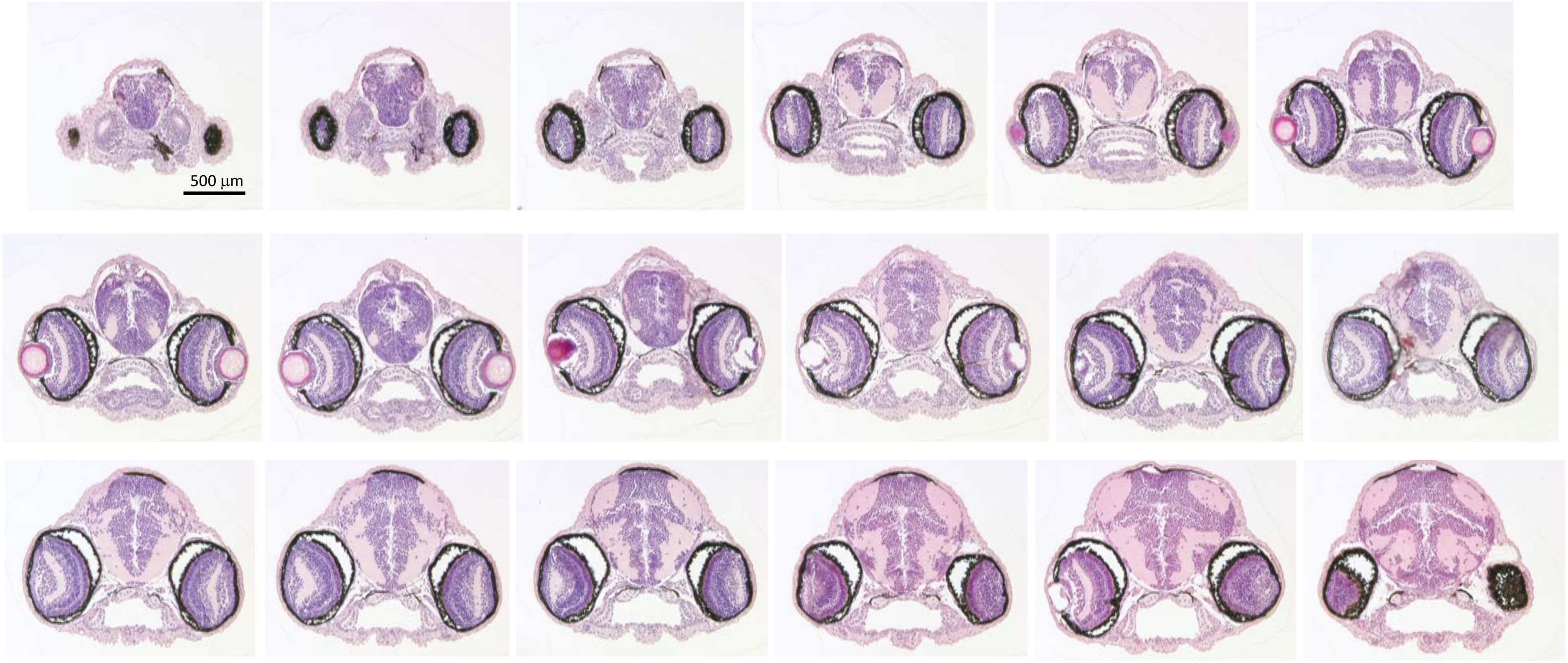

5 dpf *vars+/+*

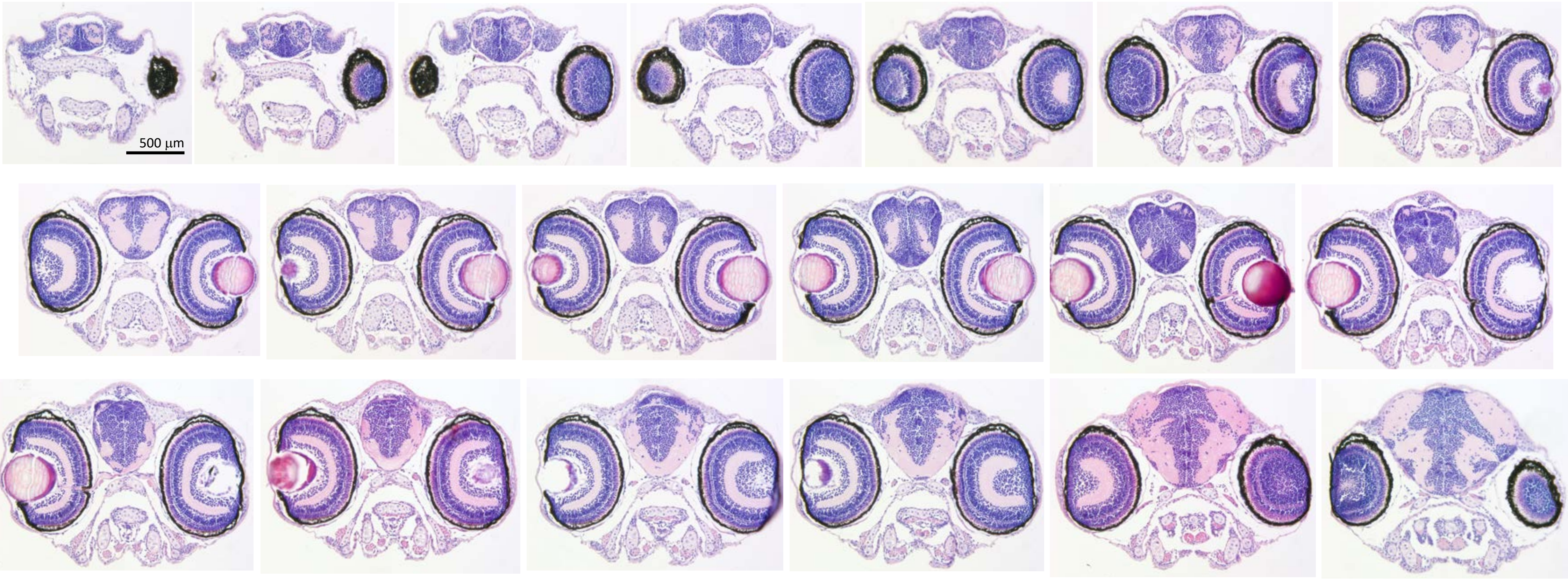

5 dpf *vars+/-*

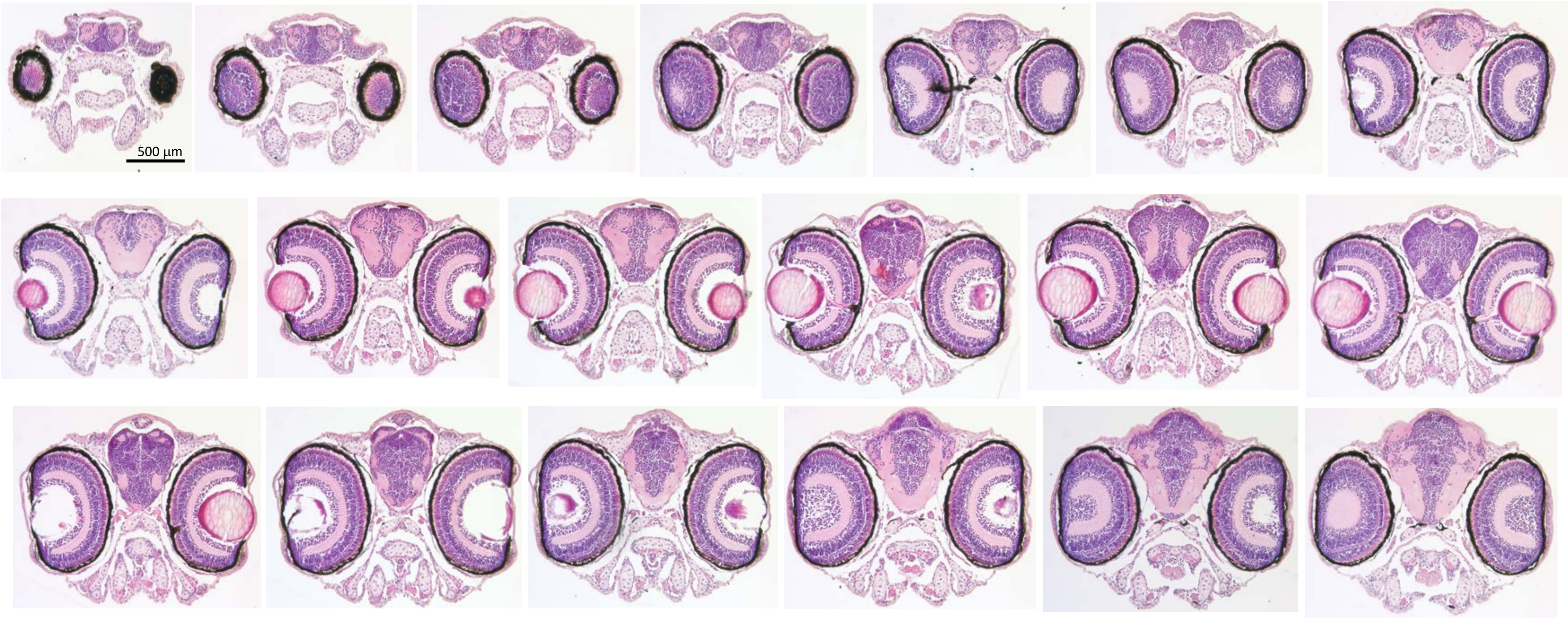

5 dpf *vars-/-*

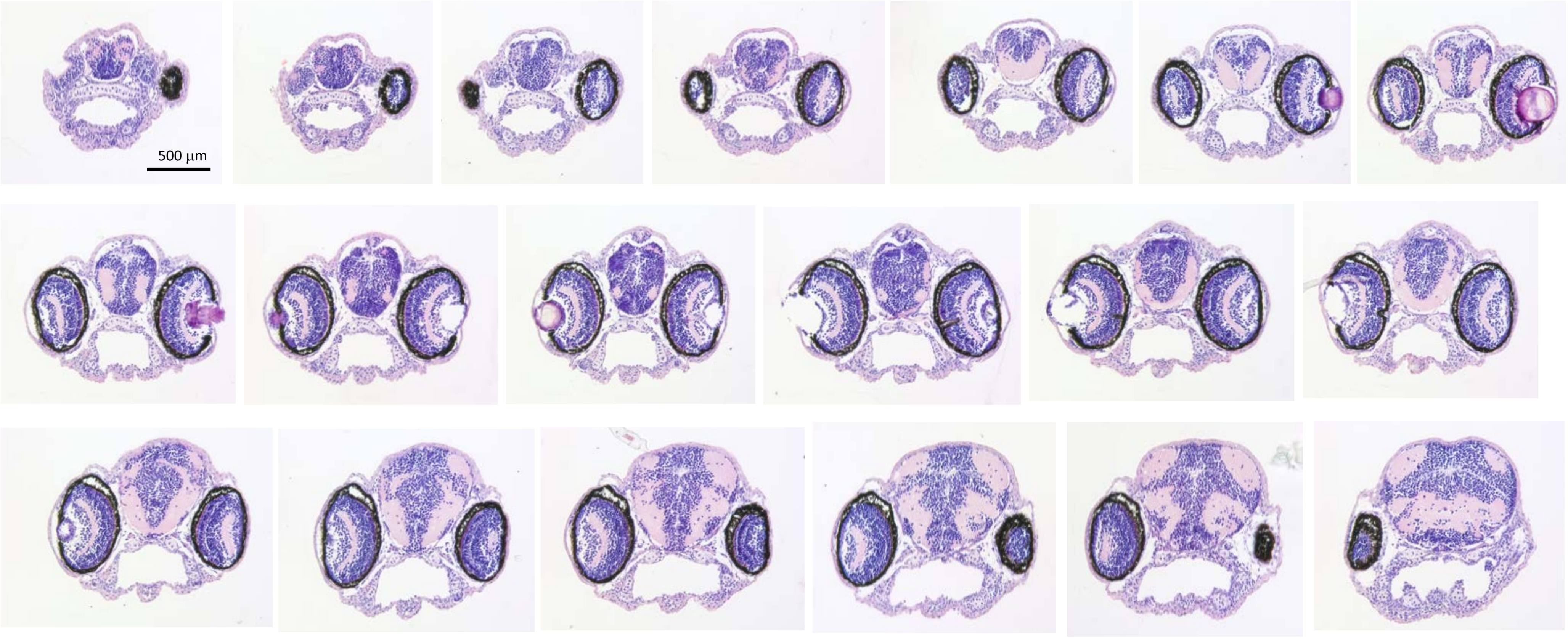

Supplement: Supplementary file 5 — Supplementary Data 2 [file 41467_2018_7953_MOESM5_ESM.pdf]
