# Supplementary material for: Biallelic VARS variants cause developmental encephalopathy with microcephaly that is recapitulated in vars knockout zebrafish
Source: Nat Commun. 2019 Feb 12;10:708. doi: 10.1038/s41467-018-07953-w (PMC6372652; doi:10.1038/s41467-018-07953-w)
Supplement: Supplementary file 1 — Supplementary Information [file 41467_2018_7953_MOESM1_ESM.docx]

***Supplementary Note 1:***

For family 1, whole-genome sequencing was performed by Complete Genomics in the context of the EUROCORES program EuroEPINOMICS on DNA of both healthy parents and the two affected children. Their service includes library preparation, massively parallel short-read sequence by ligation, sequence mapping, local *de novo* assembly, as well as structural variant calling and generation of alignment and coverage files^1^. All variants were annotated and analysed with the in-house developed GenomeComb tool^2^. Variant prioritization was done as previously described^3-5^. In brief, variants were selected based on the following criteria: (a) high quality calls (i.e. coverage >10 and exclusion of repeat regions); (b) absent in a homozygous state and frequency below 1% in control populations [data from 1000 Genomes, Exome Variant Server (EVS), Exome Aggregation Consortium, and in-house projects]; (c) predicted impact on the encoded protein (i.e. missense, nonsense, frameshift and essential splice variants) according to the RefGene annotation hg19; and (d) segregation analysis fitting an autosomal recessive model (i.e. homozygous or compound heterozygous). In addition, heterozygous variants called in both siblings and absent in the parents were selected under a parental mosaicism hypothesis. After this analysis only the compound heterozygous *VARS* variant remained. The variant was validated using direct Sanger sequencing in the respective family, including all available relatives.

***Supplementary Note 2:***

WES analysis on the proband-parent trio for family 2 was performed at Ambry Genetics (Aliso Viejo, CA). Genomic DNA extraction, exome library preparation, sequencing, bioinformatics, and data analyses were performed as previously described, taking into account possible *de novo*, x-linked and recessive inheritance^6^. Briefly, samples were prepared and sequenced using paired-end, 100 cycle chemistry on the Illumina HiSeq 2500 sequencer. Exome enrichment was performed using the IDT xGen Exome Research Panel V1.0. Data were annotated and filtered with the Ambry Variant Analyzer tool based, among others, on nucleotide and amino acid conservation, biochemical nature of the amino acid substitutions, population frequencies, and *in silico* predictions of impact on protein function^6^. Approximately 98% of the proband’s exome was covered at 20x or higher. Identified candidate alterations were confirmed using bidirectional automated fluorescence dideoxy sequencing in all available family members. This analysis resulted in one other variant than the *VARS* variant. This was a *de novo* variant in the *UBE2O* that was also seen 1 time in ExAC/gnomAD. *UBE2O* encodes a class II ubiquitin-conjugating enzyme with ubiquitous expression including high expression in the central nervous system. To date, no disease has been associated with the gene though it has been suggested to play a role in carcinogenesis and possibly other human disease^7^. This variant was classified as of uncertain significance.

| ***Gene*** | ***RefSeq ID*** | ***Alteration*** | ***Inher*** | ***ExAC*** | ***gnomAD*** | ***PolyPhen*** | ***SIFT*** | ***HGMD Disease Association*** | ***OMIM Disease*** |
| --- | --- | --- | --- | --- | --- | --- | --- | --- | --- |
| **UBE2O** | NM_022066 | c.3263G>A p.Arg1088Gln | de novo | 0,0008% | 0,0004% | probably damaging | deleterious | N/A | N/A |

***Supplementary Note 3:***

WES analysis for family 3 was performed at the Translational Genomic Research Institute (Phoenix, AZ). The affected siblings, mother, and father underwent WES. Genomic DNA was extracted from peripheral blood using the DNeasy extraction kit (Qiagen, Hilden, Germany). Genomic libraries were prepared for sequencing with the Kapa Biosystems Hyper DNA Prep Kit for Illumina Platforms (Woburn, MA, USA). Exome capture was performed with the Agilent SureSelectXT Target Enrichment Platform using Clinical Research Exome baits (Santa Clara, CA, USA). Sequencing was performed by 100 bp paired-end sequencing on a HiSeq4000 instrument (Illumina Inc., San Diego, USA). Filtered reads were aligned to the Human genome (Hg19/GRCh37) using the Burrows-Wheeler transform (BWA v.0.5.10). Reads were sorted and PCR duplicates were removed using Picard v1.79. Base quality recalibration and indel realignment were performed using the Genome Analysis Toolkit (GATK v3.1-1). Variants were jointly called with HaplotypeCaller and recalibrated with GATK, annotated with SnpEff, and selected for protein-coding events using SnpSift. Quality controls were conducted using FASTQC v0.11.5. Data was filtered against dbSNP137, 1000 Genomes, an in-house exome database of over 2,600 exomes, and then annotated with SnpEff v3.0a against Ensembl GRCh37.66 to identify novel damaging mutations. Prediction scores were loaded from dbNSFP (Database for Nonsynonymous SNP’s Functional Predictions) and used for filtering. Relatedness and gender for the nuclear family was confirmed using Plink v1.07. An annotated variant list was generated for the family and filtered for variants fitting known genetic models including heterozygous *de novo* (assuming germline mosaicism), homozygous recessive, and compound heterozygous recessive. Selected candidate variants were then prioritized based on several methods: population frequency by the ExAC and gnomAD database, prediction of protein-damaging effects by *in silico* algorithms such as ExAC’s probability of loss-of-function intolerance score (pLi), Combined Annotation Dependent Depletion (CADD), Genomic Evolutionary Rate Profiling (GERP), MutationTaster, PolyPhen2 among others, and association with human disorders with a neurological phenotype. After this filtering process, the compound heterozygous in *VARS* was the only remaining variant.

***Supplementary Note 4:***

WES analysis for families 4 en 5 was performed as reported previously^8^.

***Supplementary Note 5:***

The proband-parent trio of family 6 underwent WES at the Institute of Human Genetics, Technische Universität München, Germany. For library preparation, genomic DNA was sheared to the average size of 150 bp (Covaris) and the exome libraries prepared using SureSelect Human All Exon Kit (Agilent, 60Mb V6). The samples were sequenced on the HiSeq4000 (Illumina) platform. Mapping to the human reference genome (Hg19) was done with BWA and variant calling was performed with SAMtools and customized pearl scripts. Subsequent annotation and filtering were executed with SAMtools. Variants were filtered based on following quality parameters: coverage >20, quality >50 and not located in homopolymers >8 or tandem repeats. Only variants with a MAF < 1.0 % in control population databases ExAC, gnomAD and Exome Variant Server, seen < 1 % in the local WES database, and with predicted impact on the encoded protein (missense, nonsense, frameshift, deletions, insertions and (essential) splice site) were retained for further analysis. Remaining variants were filtered under an autosomal recessive (homozygous or compound heterozygous) and x-linked hypothesis. This analysis revealed 15 variants other than the homozygous *VARS* variant of which 7 were predicted damaging by at least PolyPhen2 or SIFT (listed below). Segregation in the healthy sibling could not be tested because DNA was not available. Careful analysis of all variants revealed the bi-allelic *VARS* variant as the likely pathogenic one.

| ***Gene*** | ***RefSeq ID*** | ***Alteration*** | ***Inher*** | ***ExAC*** | ***gnomAD*** | ***PolyPhen2*** | ***SIFT*** | ***HGMD***  ***Disease***  ***Association*** | ***OMIM Disease*** |
| --- | --- | --- | --- | --- | --- | --- | --- | --- | --- |
| *TACC2* | *NM_206862.2* | *c.4805A>G,*  *p.Glu1602Gly* | *maternal* | *0.00005788* | *0.00003255* | *benign* | *0 (D)* | *N/A* | *N/A* |
|  | *NM_206862.2* | *c.4880T>G,*  *p.Val1627Gly* | *paternal* | *0.000008255* | *0.00002167* | *benign* | *0 (D)* | *N/A* | *N/A* |
| *C6orf48* | *NM_001040438.1* | *c.56C>T,*  *p.Thr19Ile* | *hom* | *N/A* | *N/A* | *benign* | *0 (D)* | *N/A* | *N/A* |
| *SCAND3* | *NM_052923.1* | *c.1622T>A,*  *p.Val541Asp* | *hom* | *N/A* | *N/A* | *benign* | *0 (D)* | *N/A* | *N/A* |
| *ZNF142* | *NM_001105537.2* | *c.1705C>T,*  *p.Arg569Cys* | *hom* | *0.00003311* | *0.00005772* | *Possibly damaging* | *0.03 (D)* | *N/A* | *N/A* |
| *PIEZO1* | *NM_001142864.2* | *c.3901G>A,*  *p.Val1301Ile* | *hom* | 0.0001034 | *0.00004986* | *benign* | *0.01 (D)* | *N/A* | *# 616843 Lymphedema,*  *hereditary, III (AR)*  *# 194380 Dehydrated*  *hereditary*  *stomatocytosis (AD)* |
| *TET1* | *NM_030625.2* | *c.1169G>T,*  *p.Gly390Val* | *hom* | *N/A* | *N/A* | *benign* | *0.01(D)* | *N/A* | *N/A* |
| *SIRT1* | *NM_012238.4* | *c.143C>T,*  *p. Pro48Leu* | *hom* | *N/A* | *0.0001362* | *benign* | *0.02 (D)* | *N/A* | *N/A* |

***Supplementary Note 6:***

WES was performed in the proband of family 7 as previously described^9^ and yielded 53.5 million mapped reads with a mean coverage of 94 X. Following alignment and variant calling, a series of filtering steps were performed. These included removing variants which were called less than 7 X, variants not falling in coding regions and synonymous variants. The analysis further focused on recessive variants in autologous genes with a MAF < 0.1% in ExAC (Exome Aggregation Consortium, Cambridge, MA, (URL: http://exac.broadinstitute.org)) or MAF < 2% at the Hadassah in-house database (~1200 ethnic matched exome analyses). Nine variants survived this filtering but after segregation checking in the family only 4 remained, including the *VARS* variant, that were predicted to have a deleterious effect on protein function by at least PolyPhen2 or SIFT (listed below). Careful analysis of all remaining variants revealed the homozygous *VARS* variant as the best candidate.

| ***Gene*** | ***RefSeq ID*** | ***Alteration*** | ***Inher*** | ***ExAC*** | ***gnomAD*** | ***PolyPhen2*** | ***SIFT*** | ***HGMD Disease***  ***Association*** | ***OMIM***  ***Disease*** |
| --- | --- | --- | --- | --- | --- | --- | --- | --- | --- |
| *SERGEF* | NM_012139 | *c.863C>T,*  *p.Thr288Ile* | *hom* | *N/A* | *N/A* | *probably*  *damaging* | *0.005 (D)* | *N/A* | *N/A* |
| *ABCC3* | NM_003786 | *c.3179T>C,*  *p.Phe1060Ser* | *hom* | *N/A* | *N/A* | *possibly*  *damaging* | *0.008 (D)* | *No robust*  *association with*  *human phenotype* | *N/A* |
| *ARGHAP27* | NM_00128229 | *c.2126G>A,*  *p.Arg709Gln* | *hom* | *2.85 e-5* | *2.989 e-5* | *benign* | *0.017 (D)* | *N/A* | *N/A* |

***Supplementary Table 1:*** *Identified VARS variants.*

| **Fam** | **Inher** | **GRCh37/hg19** | **NM_006295.02** | **Protein effect** | **PolyPhen-2 HVAR** | **SIFT** | **VEST3 rank score** | **CADD (PHRED)** | **MAF ExAC** | **MAF gnomAD** |
| --- | --- | --- | --- | --- | --- | --- | --- | --- | --- | --- |
| **I** | comp htz | Chr.6:g.31753070G>C | c.1300C>G | p.Leu434Val | Probably damaging | Deleterious | 0.886 | 27 | - | - |
|  |  | Chr.6:g.31749338C>T | c.2464G>A | p.Gly822Ser | Probably damaging | Deleterious | 0.992 | 31 | 0.00002588 | 1.66e-5 |
| **II** | comp htz | Chr.6:g.31753412T>G | c.1199A>C | p.Gln400Pro | Benign | Deleterious | 0.439 | 23.1 | - | - |
|  |  | Chr.6:g.31753045C>T | c.1325G>A | p.Arg442Gln | Probably damaging | Deleterious | 0.911 | 35 | - | - |
| **III** | comp htz | Chr.6:g.31762763-31762776 dupGCTGGGCCACAGCC | c.219_232dup  GGCTGTGGCCCAGC | p.Leu78Argfs*35 | - | - | - | - | - | 4.274e-6 |
|  |  | Chr.6:g.31748318C>T | c.2825G>A | p.Arg942Gln | Probably damaging | Deleterious | 0.774 | 35 | - | - |
| **IV** | hom | Chr.6:g.31748712G>A | c.2653C>T | p.Leu885Phe | Probably damaging | Deleterious | 0.588 | 30 | - | - |
| **V** | hom | Chr.6:g.31747500C>T | c.3173G>A | p.Arg1058Gln | Probably damaging | Deleterious | 0.815 | 33 | 0.000008696 | 1.104e-5 |
| **VI & VII** | hom | Chr.6:g.31753401G>A | c.1210C>T | p.Arg404Trp | Probably damaging | Deleterious | 0.853 | 35 | 0.000008917 | 8.328e-6 |

All identified *VARS* variants are presented with their genomic, coding and protein position. Several *in silico* prediction scores are annotated: PolyPhen-2^10^,SIFT . The minor allele frequencies (MAF) of the variants in ExAC (<http://exac.broadinstitute.org/>) and gnomAD (<http://gnomad.broadinstitute.org>) are given. None of the variants is present more than 4x in ExAC or gnomAD and none is present in homozygous state in either of the databases. Annotations were obtained through wANNOVAR (wannovar.wglab.org)^11^.

***Supplementary Table 2:*** *Summary of predicted consequences of the identified VARS variants.*

^1^RF: Rossman Fold

^2^AC: Alpha helical anticodon binding domain

^3^Distal surface relative to tRNA binding

**Substitutions that are likely to have a direct or indirect effect on protein structure:**

| **Human variant** | **Corresponding AA**  *T. thermophilus* | **Conservation** | **Structural consequence** |
| --- | --- | --- | --- |
| *p.Leu78fs* | (not present) | n/a | NMD |
| *p.Gln400Pro* | Glu 98 | Semi: Glu, Asn, Gln, Arg | RF^1^: C-ter helix α3 |
| *p.Arg404Trp* | Arg103 | Yes, but not in Yeast | RF^1^: may help stabilize orientation of editing domain rel. to catalytic domain |
| *p.Leu434Val* | Leu 132 | Yes, only one Met | RF^1^: C-ter helix α4 |
| *p.Arg442Gln* | Arg 140 | Yes (only one Gln) | RF^1,3^: 3-10 helix α3 |
| *p.Gly822Ser* | Gly 508 | Yes (Pro in *E.coli*) | RF^1,3^: loop α12—β28 |
| *p.Leu885Phe* | (lacks insertion sequence) | Yes, only one Met | AC^2^: insertion in helix α13 |
| *p.Arg942Gln* | Trp 571 | Arg in high. Euk. | AC^2^: start of in helix α15 |
| *p.Arg1058Gln* | Lys 677 | Arg in high. Euk. | AC^2,3^: end of helix α19 |

- *p.Gln400Pro* alters a contact near the C-terminal end of helix α3. In *T. thermophilus* ValRS, the residue corresponding to the variant is Glu98, which forms a salt bridge with Arg113. The arginine is replaced by glutamate in the human enzyme, so a potential hydrogen bond between Gln Nε2 and Glu Oε1 would be eliminated by the p.Gln400Pro substitution.
- *p.Arg404Trp* substitutes an arginine that makes a hydrogen bond and ionic contact to a nearby peptide carbonyl and glutamate, respectively. The substitution of tryptophan at 404 would disrupt these interactions, while also creating a steric clash with a nearby tryptophan.
- *p.Leu434Val* shortens a side chain that is part of the hydrophobic core stabilizing the packing of helices α2, α4, and α13. Introduction of valine at this position would likely destabilize the protein, owing to weakened van der Waals interactions with adjacent hydrophobic residues.
- *p.Arg442Gln* corresponds to Arg140 in *T. thermophilus*, and this residue makes a direct hydrogen bond with a peptide carbonyl, and a weak (> 3 Å) salt bridge with a nearby aspartate. Both stabilizing contacts would be eliminated by introduction of glutamine.
- *p.Gly822Ser* is predicted to influence local structure via another mechanism. The local conformation glycine at this position (phi = -68.3°, psi = 19.9°) corresponds to a left-handed a helix that would not tolerate serine in this conformation.
- *p.Arg1058Gln* corresponds to Lys677 in the *T. thermophilus* enzyme. This residue makes weak hydrogen bonds to a tyrosine and a nearby backbone carbonyl. Both interactions would likely be lost in the p.Arg1058Gln variant.

**Substitutions that are more likely to affect substrate recognition/ tRNA binding:**

- *p.Arg942Gln* corresponds to Trp571 in the *T. thermophilus* ValRS complex. In the complex, the Nη hydrogen of the adjacent Arg570 makes direct hydrogen bonds with the O2 atoms of C11 and C25, respectively. The tryptophan makes a stacking interaction with the backbone. In the higher eukaryotes, Arg570 is replaced by asparagine, and the adjacent residue switches from asparagine to arginine. Thus, among all mutants, p.Arg942Gln is the best candidate for a substitution that directly affects the binding of a substrate, based on the assumption that substitution of Arg with Gln would reduce tRNA affinity.
- *p.Leu885Phe* is located in an insertion in helix α13 that is present in *E. coli* and most eukaryotes but is missing in *T. thermophilus* ValRS. Inspection of the structure of the complex suggests that the insertion in which p.Leu885Phe resides, is potentially close to the N4 of the tRNA anticodon nucleotide C38 in the bound complex. The addition of a bulky non-polar residue like phenylalanine into this loop may affect one or more direct contacts to anticodon functional groups.

***Supplementary Table 3****: Primers used for different experiments.*

| ***Orientation*** | ***Primer sequence (5’*** *🡪* ***3’)*** |
| --- | --- |
| a. **Primers used for RT-qPCR cell line patient 4 and 5** | |
| Amplicon 1 F (ex1) | GGAACTAGGGTCACCTGGAG |
| Amplicon 1 R (ex1) | GTGGAAAAACTGGAGGGAGA |
| b. **Primers used for complementation assays in yeast** | |
| *VAS1* gene amplif. F | CATTTAGAATTTCAAATATATCC |
| *VAS1* gene amplif. R | GATTATAATATACCACAACATGC |
| L434V mutagenesis F | AACCAAATTCAAAAAGTGGGGGCTTCTTATG |
| L434V mutagenesis R | CATAAGAAGCCCCCACTTTTTGAATTTGGTT |
| R442Q mutagenesis F | CTTATGATTGGAGCCAAGAAGCTTTCACTTTG |
| R442Q mutagenesis R | CAAAGTGAAAGCTTCTTGGCTCCAATCATAAG |
| G822S mutagenesis F | TCTATGTTGGAAACTAGTTGGGATATTCTTT |
| G822S mutagenesis R | AAAGAATATCCCAACTAGTTTCCAACATAGA |
| L885F mutagenesis F | ATTAAGTTGGATGATTTCCATGCAAAATTATTA |
| L885F mutagenesis R | TAATAATTTTGCATGGAAATCATCCAACTTAAT |
| c. **Primers used for making the WISH probe.** | |
| Forward | GAAAGCCAAACAGGGACAGA |
| Reverse | CTTTCTTGGCCGTCAGTTTC |
| d. **Primers used for genotyping of *vars* knockout CRISPR line.** | |
| Forward | AGAACCAGAACCAAACTTGTGTGTG |
| Reverse | ATGCCGGTGATGACGTCCAG |
| e. **Primers used for RT-qPCR for CRISPR knockout line.** | |
| *vars* forward | AGAGTCTTCTACCCGGGC |
| *vars* reverse | CCATCATGACCATTCGAGCA |
| *18s* forward | TCGCTAGTTGGCATCGTTTATG |
| *18s* reverse | CGGAGGTTCGAAGACGATCA |
| f. **Primers used for genotyping of *vars*^Hi558Tg^ knockout line.** | |
| forward (in rvi) | GCACGTTCTTCTGTTATTGCGTC |
| reverse (in rvi) | CAAACCTACAGGTGGGGTCTTTC |
| forward (in cds) | TTTGTGTTGGTCAGTAAAACTGC |
| reverse (in cds) | GACTGCCTGAGACTGTCAGATG |
| g. **Primers used for RT-qPCR for *vars*^Hi558Tg^ knockout line.** | |
| *vars* primer 1 | CATTGCCGAAGTAAGCGTTG |
| *vars* primer 2 | AGGGTGGGAAGAGATGTAGAG |
| *ef alpha* primer 1 | CACCAGGACACAGAGACTTC |
| *ef alpha* primer 2 | GTGTTTGCCCGTTCTTTGAG |
| *18s* primer 1 | CACTGAGGATGAGGTTGAGAG |
| *18s* primer 2 | TTGTCCAGACCATTAGCAAGG |
| h. **Primers used for *VARS* site directed mutagenesis for rescue experiments zebrafish** | |
| Q400P mutagenesis F | GAAGCTATGGCGTGAGCCGGGACTGA |
| Q400P mutagenesis R | TCAGTCCCGGCTCACGCCATAGCTTC |
| R942Q mutagenesis F | ACCTGGATGTGAACCAGATACTGGGTTACC |
| R942Q mutagenesis R | GGTAACCCAGTATCTGGTTCACATCCAGGT |
| R1058Q mutagenesis F | CGTTGGCCTGCAGCTGCTCTCA |
| R1058Q mutagenesis R | TGAGAGCAGCTGCAGGCCAACG |

Summary of primer sequences for primers used in the functional experiments included in this study. All sequences are given in 5’ to 3’ direction. Rvi: retroviral insertion, cds: coding sequence.

***Supplementary Figure 1:*** *Sequence alignments of the selected regions in Thermus thermophilus VARS protein coding sequence and its orthologues.*

The top row represents secondary structures derived from the structure of the ValRS-tRNA^Val^ from *Thermus.* The rows below show the sequence alignment of amino acid sequences of *Thermus*, yeast*,* worm, *E. coli*, fruit fly, zebrafish, mouse and human. The patient mutations, highlighted in blue frame, are generally conserved among vertebrates. Red color depicts strict identity, whereas yellow color indicates similar residues. The alignments were generated by Clustal Omega and annotated in the protein ENDscript.

***Supplementary Figure 2:*** Immunocytochemistry: co-staining for VARS and endoplasmic reticulum, mitochondrial and Golgi-complex markers in control fibroblast cells.

Figure showing ICC staining in fibroblast of VARS (green) and markers (red) for endoplasmic reticulum (KDEL), the Golgi-complex (Golgin) and mitochondria (TOMM20). The nucleus is stained with Hoechst 33342 (blue). From left to right there is an overlay image showing both staining markers and the nucleus, a zoomed image, and then separately a panel showing VARS and the respective organelle marker. VARS co-localizes with ER but not with the Golgi complex or mitochondria.

***Supplementary Figure 3:*** *Spatiotemporal expression patterns of vars by whole-mount RNA in situ hybridization.*

Images of *vars* expression in developing AB embryos and larvae of (**a, a’, h**) 6 hpf, (**b, b’, i**) 18 hpf, (**c, c’**) 24 hpf, (**d, d’, j**) 36 hpf, (**e, e’**) 48 hpf, (**f, f’, k**) 72 hpf, and (**g, g’**) 4.5 dpf; (**a-g**) with anti-sense RNA probe, (**h-k**) with sense RNA probe as negative control. **a-k**, lateral view; **a’-g’**, dorsal view. Rostral to left. Although not detectable during gastrulation stage (**a**), from 18 hfp *vars* is almost ubiquitously expressed throughout the whole body (**b,c**). As development progresses, it becomes restricted to the brain regions, highly enriched in the midbrain, midbrain-hindbrain boundary, and hindbrain (**d-g**), as well as other organs such as branchial arches, liver, pancreas, and intestine. BA, Branchial Arches; H, Hindbrain; HICM, Hematopoietic Intermediate Cell Mass; IB, Intestinal Bulb; Ints, Intestine; L, Lens; Liv, Liver; MBH, Midbrain-Hindbrain boundary; M, Midbrain; Pan, Pancreas; PF, Pectoral Fin; R, Retina. Scale bars = 200 μm.

***Supplementary Figure 4:*** *Generation of vars CRISPR knockout zebrafish line using CRISPR/Cas9 system.*

(**a**) Scheme illustrating the process of generating *vars* CRISPR line. Single cell-stage wild type (WT) embryos were injected with *vars* sgRNA and Cas9 mRNA. Injected embryos were raised till adulthood and among them an F0 founder was selected that was subsequently outcrossed with WT zebrafish. F1 generation embryos were raised till adulthood, fin clipped and sequenced. Zebrafish carrying the same mutation were identified and pooled together. All experiments were performed on embryos coming from F2 or F3 progeny. (**b**) Confirmation by Sanger sequencing of the 4 bp deletion (GGGC) generated using CRISPR/Cas9 method. Chromatograms of *vars*+/+, *vars*+/- and *vars*-/- are depicted.

***Supplementary Figure 5:*** *Morphological characterization of vars-/- larvae and analysis of the apoptosis.*

(**a**) Representative lateral and dorsal bright-field images of 1, 3 and 5 dpf *vars*+/+, *vars*+/- and *vars*-/- larvae (scale bar, 500 μm). Small eye and periocular oedema, as well as pericardial oedema are marked with red arrows. (**b**) Assessment of apoptosis by immunohistochemistry to active caspase-3. Bar graph showing the percentage of apoptotic cell (DAB-positive cells) out of the total haematoxylin-stained cell number in selected corresponding brain areas of 2-5 dpf *vars* +/+, *vars* +/- and *vars* -/- (left panel). Representative images of paraffin-embedded coronary sections of 5 dpf *vars*+/+, *vars*+/- and *vars*-/- larvae stained for active caspase-3 (right panel). Chromogenic substrate (DAB) for HRP conjugated secondary antibody is transformed by the active enzyme in a colorized product (brown), indicating the presence of programmed cell death in a given brain spot. Hematoxylin counterstain (blue).

One-way ANOVA with Tukey's multiple comparisons test, and significant values are noted as *p<0.05, **p<0.01 and ***p<0.001. All experiments were performed in triplicate. All error bars represent SD.

***Supplementary Figure 6:*** *Morphological and behavioural characterization of vars^Hi558Tg^ larvae****.***

(**a**) Kaplan-Meier survival curve of *vars*^Hi558Tg+/+^ (n=64), *vars*^Hi558Tg+/-^ (n=108) and *vars*^Hi558Tg-/-^ (n=67) larvae. (**b**) Graph illustrating changes in loss of posture and touch response of *vars*^Hi558Tg-/-^ larvae throughout the life span. Only the surviving larvae are included (n=67). (**c**) Representative lateral and dorsal bright-field images of 5 dpf *vars*^Hi558Tg+/+^, *vars*^Hi558Tg+/-^ and *vars*^Hi558Tg-/-^ larvae (scale bar, 500 μm). Pericardial oedema, small eye and periocular swelling around the eye are marked with arrows. (**d-f**) Comparison of the individual measurements for head size (**d**), brain size (**e**) and eye size (**f**) for *vars*^Hi558Tg+/+^, *vars*^Hi558Tg+/-^ and *vars*^Hi558Tg-/-^ at 3 dpf (+/+ n=14, +/- n=17 and -/- n=11) and 5 dpf (+/+ n=7, +/- n=17 and -/- n=15). All error bars represent SD. (**g**) Behavioural activity (average total movement) of *vars*^Hi558Tg+/+^ (n=48), *vars*^Hi558Tg+/-^ (n=121) and *vars*^Hi558Tg-/-^ (n=57) larvae from 3 to 5 dpf expressed in actinteg units (300 s time interval). (**h**) Representative recording from optic tectum of 4 dpf *vars*^Hi558Tg-/-^ larva displaying polyspiking discharges. Top trace represents typical pattern of epileptiform activity. Bottom trace shows magnification of the epileptiform event. (**i**) RT-qPCR data demonstrating the expression of *vars* in *vars*^Hi558Tg+/+^, *vars*^Hi558Tg+/-^ and *vars*^Hi558Tg-/-^ larvae from 3 to 5 dpf.

In **a**, log-rank (Mantel-Cox) test was used, in **d-g** and **i** one-way ANOVA with Tukey's multiple comparisons test, and significant values were noted as *p<0.05, **p<0.01, ***p<0.001 and ****p<0.0001. All experiments were performed in triplicate. All error bars represent SD.

***Supplementary Figure 7****: Uncropped Western blots.*

Original (uncropped) blots probed for VARS protein (left) and tubulin (loading control) (right). A non-specific band was observed at ~52 kDa.

***Supplementary references:***

1.	Drmanac, R. *et al.* Human genome sequencing using unchained base reads on self-assembling DNA nanoarrays. *Science* **327**, 78-81 (2010).

2.	Reumers, J. *et al.* Optimized filtering reduces the error rate in detecting genomic variants by short-read sequencing. *Nat Biotechnol* **30**, 61-8 (2011).

3.	Schubert, J. *et al.* Mutations in STX1B, encoding a presynaptic protein, cause fever-associated epilepsy syndromes. *Nat Genet* **46**, 1327-32 (2014).

4.	Hardies, K. *et al.* Recessive loss-of-function mutations in AP4S1 cause mild fever-sensitive seizures, developmental delay and spastic paraplegia through loss of AP-4 complex assembly. *Hum Mol Genet* **24**, 2218-27 (2015).

5.	Hardies, K. *et al.* Recessive mutations in SLC13A5 result in a loss of citrate transport and cause neonatal epilepsy, developmental delay and teeth hypoplasia. *Brain* **138**, 3238-50 (2015).

6.	Farwell, K.D. *et al.* Enhanced utility of family-centered diagnostic exome sequencing with inheritance model-based analysis: results from 500 unselected families with undiagnosed genetic conditions. *Genet Med* **17**, 578-86 (2015).

7.	Hormaechea-Agulla, D., Kim, Y., Song, M.S. & Song, S.J. New Insights into the Role of E2s in the Pathogenesis of Diseases: Lessons Learned from UBE2O. *Mol Cells* **41**, 168-178 (2018).

8.	Karaca, E. *et al.* Genes that Affect Brain Structure and Function Identified by Rare Variant Analyses of Mendelian Neurologic Disease. *Neuron* **88**, 499-513 (2015).

9.	Ta-Shma, A. *et al.* Congenital valvular defects associated with deleterious mutations in the PLD1 gene. *J Med Genet* **54**, 278-286 (2017).

10.	Adzhubei, I.A. *et al.* A method and server for predicting damaging missense mutations. *Nat Methods* **7**, 248-9 (2010).

11.	Yang, H. & Wang, K. Genomic variant annotation and prioritization with ANNOVAR and wANNOVAR. *Nat Protoc* **10**, 1556-66 (2015).
